# The prognostic value of intratumoral and peritumoral tumor-infiltrating FoxP3+Treg cells in of pancreatic adenocarcinoma: a meta-analysis

**DOI:** 10.1186/s12957-021-02420-1

**Published:** 2021-10-16

**Authors:** Lingyu Hu, Mingyuan Zhu, Yiyu Shen, Zhengxiang Zhong, Bin Wu

**Affiliations:** Department of Hepatobiliary Surgery, The Second Affiliated Hospital of JiaXing University, Jiaxing, 314000 Zhejiang China

**Keywords:** FoxP3+Treg cells, Pancreatic cancers, Prognostic

## Abstract

**Background:**

Tumor-infiltrating lymphocytes (TILs) are major participants in the tumor microenvironment. The prognostic value of TILs in patients with pancreatic cancer is still controversial.

**Methods:**

The aim of our meta-analysis was to determine the impact of FoxP3+Treg cells on the survival of pancreatic cancer patients. We searched for related studies in PubMed, EMBASE, Ovid, and Cochrane Library from the time the databases were established to Mar 30, 2017. We identified studies reporting the prognostic value of FoxP3+Treg cells in patients with pancreatic cancer. Overall survival (OS) and disease-free survival (DFS)/progression-free survival (PFS)/relapse-free survival (RFS) were investigated by pooling the data. The pooled hazard ratios (HRs) with 95% confidence intervals (95% CI) were used to evaluate the association between FoxP3+Treg cells and survival outcomes of pancreatic cancer patients. A total of 972 pancreatic cancer patients from 8 studies were included in our meta-analysis.

**Results:**

High levels of infiltration with FoxP3+Treg cells were significantly associated with poor OS (HR=2.13; 95% CI 1.64–2.77; *P*<0.05) and poor DFS/PFS/RFS (HR=1.70; 95% CI 1.04 ~ 2.78; *P*< 0.05). Similar results were also observed in the peritumoral tissue; high levels of FoxP3+Treg cells were associated with poor OS (HR =2.1795% CI, CI 1.50–3.13).

**Conclusion:**

This meta-analysis indicated that high levels of intratumoral or peritumoral FoxP3+Treg cell infiltration could be recognized as a negative factor in the prognosis of pancreatic cancer.

## Introduction

Pancreatic cancer, specifically pancreatic ductal adenocarcinoma (PDAC), is one of the most malignant cancers, with a 5-year survival rate of only 7% [1]. In the majority of patients who have been diagnosed with local or distant metastasis, the rate of surgical resection is only 20% [2] and even in the case of surgical resection, metastasis, and recurrence often occu r[3]. A large number of studies suggest that the tumor microenvironment may regulate the biological behavior of tumors [4] and because of the large number of interstitial components in pancreatic tumor tissue [5], these components play an important role in tumorigenesis, progression, and metastasis. Tumor-infiltrating lymphocytes (TILs) are one of the major participants in the tumor microenvironment [6]. The cytokines secreted by TILs are the main component of the tumor immune microenvironment, and these cytokines play a major role in tumor immune regulation.

TILs have been reported in a variety of tumors, including pancreatic cancer, but the mechanism of interaction between TILs and the tumor is very complex and still unclear. The previous studies are still controversial. Previous studies have shown that CD8+ T cell infiltration of tumors is a beneficial factor in patients with colorectal cancer [7, 8], ovarian cancer [9], lung cancer [10], and pancreatic cancer. It is confirmed that the role of CD4+ T cell infiltration is more complex in tumor progression. Th1 (helper T cell 1) cells are considered to be a prognostic factor, and Th17 (helper T cell 17) [11] and Th2 (helper T cell 2) [12] cells are considered to have a tumorigenic effect.

Regulatory T cells (Treg cells) are a specific class of CD4+ T cells that are thought to promote tumor growth and invasion by inhibiting the host’s immune response and pro-inflammatory responses [13]. FoxP3 is one of the most specific markers of Treg cells in tumors [14]. FoxP3 is a forkhead box transcription factor that contains a domain, which can bind with DNA, and is thought to inhibit target gene expression. Previous studies have shown that higher levels of FoxP3+Treg cells indicate a poor prognosis in cancer patients, but this idea has recently been challenged to suggest that high levels of tumor-infiltrating FoxP3+Treg cells are not always associated with poor prognosis but can improve survival time in some types of tumors [15, 16]. This is inconsistent with the primary hypothesis that FoxP3+Treg cells suppress anti-tumor immunity. Interestingly, this conclusion is entirely inconsistent in some tumors [8, 16], especially studies reported in tumors such as hepatocellular cancer and colorectal cancer. Most studies in pancreatic cancer suggest that high levels of FoxP3+Treg cells are a poor prognostic factor, but there are also reports of no predictive value.

In conclusion, we found that there was a need to summarize a large sample size of tumors to determine the association between tumor-infiltrating FoxP3+Treg cells and prognosis in pancreatic cancer in order to gain insight into whether FoxP3+Treg cells can provide useful guidance for the biological treatment of pancreatic cancer.

## Materials and methods

This meta-analysis was carried out following the guidelines of the Systematic Reviews and Meta-Analyses (PRISMA) [17] and the guidelines of Observational Studies in Epidemiology (MOOSE) [18].

### Literature search strategy

The literature was searched using PubMed, EMBASE, Ovid, and Cochrane Library (last update by Mar 30, 2017). Keywords for the search strategy were “(FoxP3 lymphocytes OR FoxP3 regulatory T cell OR FoxP3 TIL OR FoxP3 Treg OR FoxP3 tumor-infiltrating lymphocytes OR FoxP3+ T cell OR CD4+CD25+ lymphocytes OR CD4+CD25+ regulatory T cell OR CD4+CD25+ TIL OR CD4+CD25+Treg OR CD4+CD25+ tumor-infiltrating lymphocytes OR CD4+CD25+T cell OR CD4+CD25+Foxp3+T cell) AND (Pancreatic Neoplasm OR Pancreatic Cancer OR Pancreatic tumor)” (all fields). First, titles and abstracts were reviewed to identify studies that examined the association between FoxP3+Treg cell expression and survival outcomes, such as overall survival (OS) or indicators of recurrence (disease-free survival, DFS; progression-free survival, PFS; relapse-free survival, RFS). We also hand-searched the references of the reported studies or reviews. If the original data of the study could not be obtained from the literature, we tried to contact the author to obtain the data. If full-text duplication occurs, one of the manuscripts will be deleted.

### Selection criteria

Two authors were responsible for the independent selection and evaluation of studies from the databases. The following criteria were used for the studies included in our meta-analysis: (1) the study object must be humans; (2) the study must report the association between FoxP3+Treg cells (also including CD4+CD25+TILs) and the survival outcomes of pancreatic cancer patients; (3) the reported expression level of FoxP3+Treg cells included the tumor bed but not peripheral circulating lymphocytes, and the method of detecting this marked was immunohistochemical; and (4) the study included sufficient data to determine an estimate for the hazard ratio (HR) and a 95% confidence interval (95% CI). The following were exclusion criteria for studies: (1) TILs had other markers in addition to FoxP3+ (CD4+CD25+) markers; (2) if more than one study included data from the same patient cohort, only the most suitable study was selected; and (3) some types of literature such as reviews, letters, case reports, and conference abstracts were excluded.

### Data extraction and quality assessment

Data were independently extracted from the included studies by two investigators. Discrepancies were resolved by consensus. The parameters of all the studies were collected, including the name of the first author, publication year, sample size, tumor stage, the follow-up period, distribution site of FoxP3+Treg cells, the cut-off definition, HR of FoxP3+Treg cells for OS, DFS, PFS, and RFS as well as the corresponding 95% CIs. If the HR and CIs were not provided, the total number of observed death events and the number of patients in each experimental group were extracted to calculate the HR and 95% CIs. The results of the multivariate analysis were selected to obtain the HR when both the multivariate and univariate analyses were provided. The quality of each study was independently assessed by two researchers according to the Newcastle Ottawa Quality Assessment Scale (NOS) [19] as was done in McShane et al. [20]. The quality assessment scores ranged from 0 to 9 and studies with a score ≥ 6 were regarded as high quality.

### Statistical analysis

For time-to-event outcomes, we used the pooled HR and its 95% CI to evaluate the impact of higher levels of FoxP3+Treg cells on the OS and DFS/PFS/RFS of patients with pancreatic cancer. Most of the included studies provided an HR and 95% CIs, which we extracted directly from the papers. When Kaplan-Meier curves were provided rather than the HR, the HR was estimated indirectly from the curves using the software Engauge Digitizer Version 4.1 (http://digitizer.sourceforge.net/) by the method described in Parmar et al. [21]. The HR from each study provided an estimate of the ratio of the HR for a high level over a low-level FoxP3+Treg cell infiltration. Thus, high levels of FoxP3+Treg cells implied a poor prognosis when the HR>1, whereas an HR<1 implied a good prognosis.

Heterogeneity across the studies was assessed using a chi-square-based *Q* statistical test and was quantified using the *I*^2^ statistic, with an *I*^2^ value>50% indicating substantial heterogeneity. When there was no statistically significant heterogeneity (*I*^2^ value<50%), a pooled effect was calculated with a fixed-effects model. Otherwise, the pooled statistics were calculated using the free-effects model. Subgroup analyses were carried out according to the locations of FOXP3+T infiltration (intratumoral or peritumoral).

Publication bias was examined by visually inspecting funnel plots and was quantified using Egger’s or Begg’s regression model, in which a *P* value of <0.10 was considered to be significant. In addition, a sensitivity analysis was performed to examine the stability of the pooled results.

The statistical analyses were conducted using STATA (version 12.0). All *P* values < 0.05 were considered to be statistically significant.

## Results

### Study selection and characteristics

**Fig. 1:**
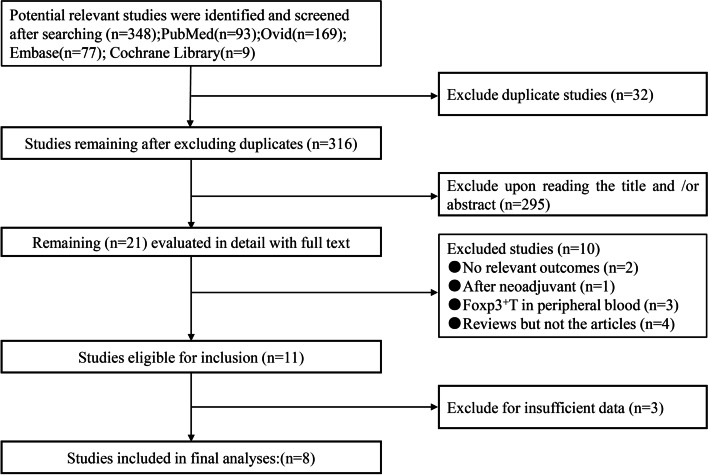
Selection of studies

As shown in the selection process of the eligible studies (Fig. 1), a total of 348 studies were initially obtained from the abovementioned databases using the search strategy described above. From candidate studies following exclusions, we screened the studies and obtained 8 studies that met the inclusion criteria. The main characteristics of the included studies are presented in Table 1. A total of 972 patients with pancreatic cancer were enrolled in these studies. The individual sample size ranged from 40 to 212 (mean 121.5). These studies were conducted in 4 countries (the UK, China, Japan, and Switzerland). Survival data were extracted from the Kaplan-Meier curves for two studies (studies 3 and 14). Three studies [29–31] had published insufficient data and did not provide an HR with a 95% CI or data sufficient for estimating the HR. We tried to contact the corresponding authors of those studies for additional information, but neither of them responded. Two studies also included intratumoral and peritumoral tissues with their corresponding survival data, and eight studies used OS as a prognostic indicator to evaluate the significance of FoxP3+Treg cells in intratumoral tissues. Three studies used both OS and PFS as indicators, and only one study used both OS and DFS as indicators. Overall, the included studies in the meta-analysis were considered to be high quality; six studies scored 8, one study scored 7, and one study scored 6.

**Table 1:** Characteristic of included studies: FoxP3+Treg cells and survival outcome

study	M/F	Tumor stage	Marker	Location	Number(H/L)	Cutoff	Outcome
**Wartenberg M 201 5**[22]	**120(64/53)**	**3/98/4/10 (I/II/III/IV)**	**FoxP3**^**+**^	**I**	**53/55**	**Median normalized value**	**OS**
				**P**	**50/58**	**Median normalized value**	**OS**
**Tang Y 201 4**[23]	**45(28/17)**	**34/11 (I+II/III+IV)**	**CD4**^**+**^**CD25**^**+**^**Foxp3**^**+**^	**I**	**22/23**	**Median value**	**OS**
**Wang X 201 5**[24]	**120(66/54)**	**28/92 (I+II/III+IV)**	**FoxP3**^**+**^	**I**	**76/44**	**Not mentioned**	**OS**
							**PFS**
**Ino Y 201 3**[4]	**212(132/80)**	**2/188/0/22 (I/II/III/IV)**	**FoxP3**^**+**^ **CD4**^**+**^		**106/64**	**Not mentioned**	**OS**
							**PFS**
**Liu L 201 6**[25]	**92(68/24)**	**25/41/26 (I/II/III)**	**FoxP3**^**+**^	**I**	**44/48**	**Median value**	**OS**
							**PFS**
				**P**	**36/56**	**Median value**	**OS**
							**PFS**
**Hiraoka N 200 6**[26]	**198(114/84)**	**16/10/74/98 (I/II/III/IV)**	**FoxP3**^**+**^	**I**	**104/94**	**Not mentioned**	**OS**
**Diana A 201 6**[27]	**145(77/68)**	**88/57 (I+II/III+IV)**	**FoxP3**^**+**^	**I**	**38/107**	**Not mentioned**	**OS**
				**I**			**DFS**
**Zhang K 201 5**[28]	**40(26/14)**	**4/16/0/6 (I/II/III/IV)**	**FoxP3**^**+**^	**I**	**24/16**	**Not mentioned**	**OS**

*I* Intratumoral; *P* Peritumoral; *H* high level; *L* low level

### Intratumoral FoxP3+Treg cell level and prognosis of pancreatic cancer

**Fig. 2:**
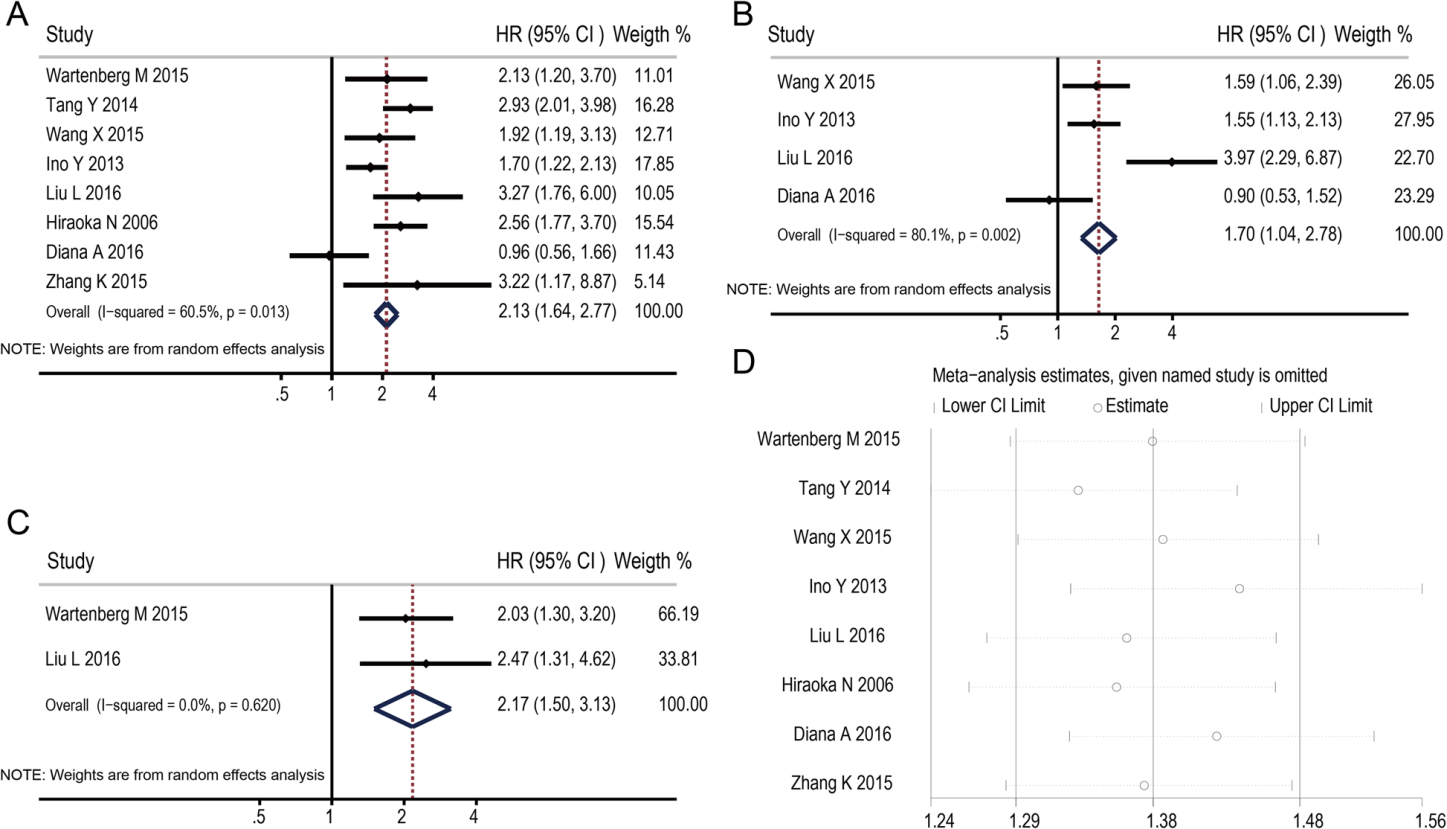
Forest plots of the hazard ratio (HR) for the association of FoxP3+Treg cell level with survival in pancreatic cancer (**A**, **B**, **C**) and the sensitivity analysis plots of OS (**D**). Forest plots of HR for the association between intratumoral FoxP3+Treg cell level and OS (**A**); Forest plots of HR for the association between intratumoral FoxP3+Treg cell level and indicators of recurrence (DFS/PFS/RFS) (**B**); Forest plots of HR for the association between peritumoral FoxP3+Treg cell level and OS (**C**); The results of the sensitivity analysis plots for OS showed that the association did not change significantly after removing any study

We pooled OS and recurrence indicators (DFS/PFS/RFS) to assess the impact of intratumoral FoxP3+ T levels on the prognosis of pancreatic cancer. Eight studies evaluated the relationship between intratumoral FoxP3+ T and OS. The pooled HR is 2.13 (95% CI 1.64–2.77; *P*<0.05) (Fig. 2A). It indicated that a higher FoxP3+ T level was statistically related to a poor OS rate. Four studies assessed the association between FoxP3+ T and recurrence indicators (DFS/PFS/RFS). The pooled HR is 1.70 (95% CI 1.04 ~ 2.78; *P*< 0.05) (Fig. 2B) which indicated that FoxP3+ T level was also correlated with the recurrence of pancreatic cancer.

### Peritumoral FoxP3+Treg cells level and prognosis of pancreatic cancer

Two studies in each outcome panel investigated FoxP3+Treg cell infiltration in peritumoral tissues; the pooled HR for the two studies for OS was 2.17 (95% CI 1.50–3.13) (Fig. 2C). However, these results should be interpreted with caution because of the small number of contributing studies and the significant evidence of heterogeneity between studies (chi-squared = 0.25; *P* = 0.620).

### Sensitivity analysis

The influence of each study on the pooled HR of survival outcomes was evaluated by repeating the meta-analysis while removing each study sequentially. The results of sensitivity analysis showed that the HR did not change significantly after removing any study (Fig. 2D).

### Publication bias

**Fig. 3:**
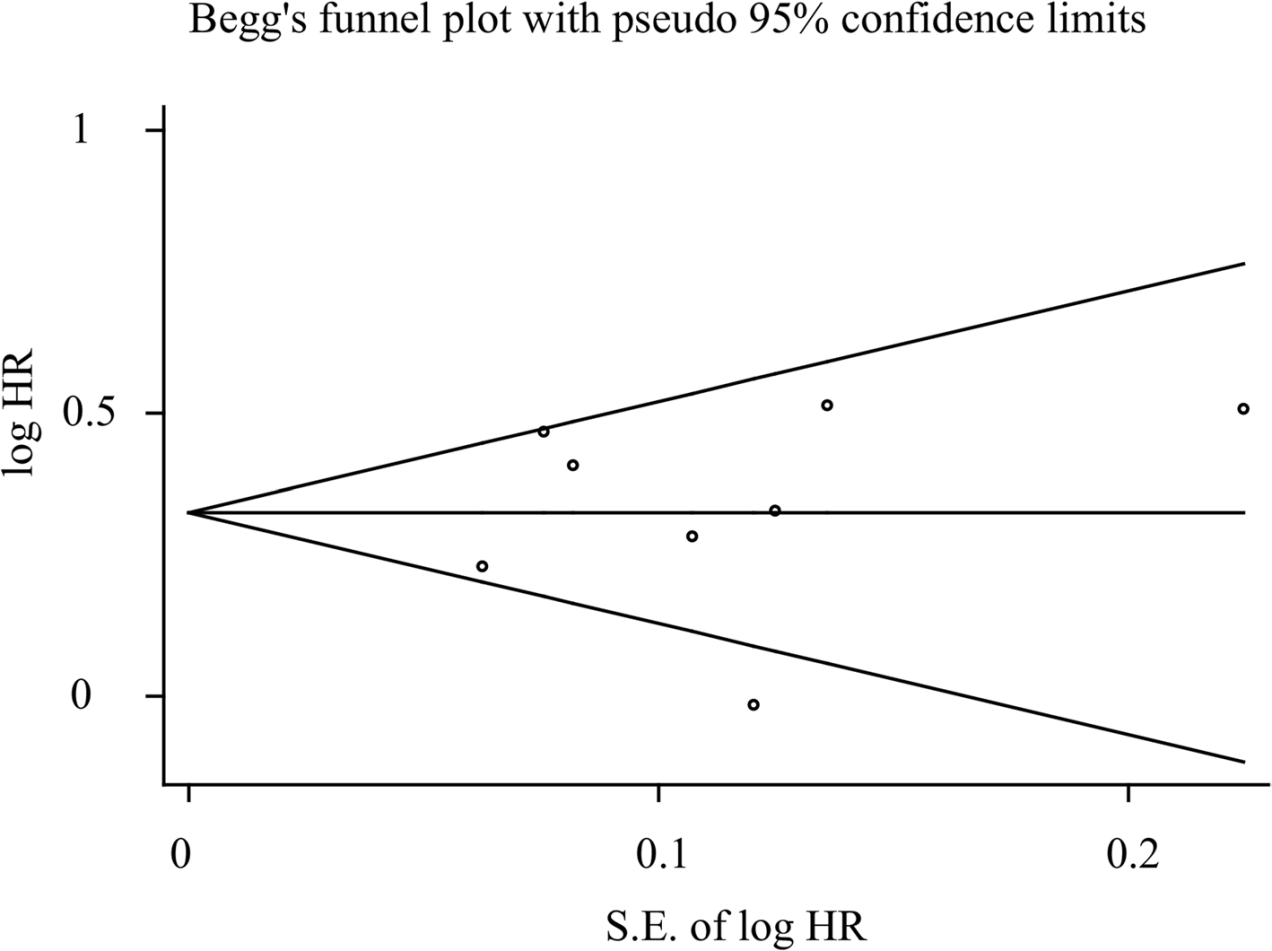
Funnel plots of the relationship between the size of the effect in individual studies and the precision of the study estimate (log HR, vertical axis; S.E. of log HR, horizontal axis) for FoxP3+Treg cells

Begg’s test indicated no publication bias after assessing the funnel plot for the studies included in our meta-analysis (Fig. 3). The *P* value =0.808>0.10, which means there is no significant publication bias in the studies that used OS as a survival outcome.

## Discussion

Anti-tumor immunity depends mainly on the immune cells in vivo. Tumor tissues are often infiltrated by immune cells, mainly lymphocytes [6]. Previous studies [32, 33] considered the hypothesis that lymphocytes attack tumor cells; however, tumor-infiltration lymphocytes have also been shown to promote tumor metastasis, resulting in poor prognosis of pancreatic cancer patients [34]. Different types of lymphocytes and different locations of tumor-infiltrating lymphocytes indicate the different survival outcomes. Treg cells, an integral component of tumor-infiltrating lymphocytes, have been widely studied recently. Treg is thought to play an important role in maintaining immune balance and immune tolerance in the body [35].

FoxP3+, the most representative marker of Treg cells, plays a critical role in immune tolerance and the suppression of anti-tumor immunity [36–38]. However, some studies [39] have shown that activated T cells can express FoxP3+, and it was also thought to not be the determine marker of Treg cells. A majority of studies revealed that the level of FoxP3+Treg cells had a negative impact on the prognosis of pancreatic cancer [23, 29]. However, other studies showed that Foxp3+Treg cells also play a dual function of inhibiting or promoting in different tumors [40]. The accurate significance of the prognosis is still unclear, and the information is limited. However, until now, there has been no meta-analysis of the prognostic significance of TILs in pancreatic cancer. Furthermore, no meta-analysis has been undertaken to evaluate FoxP3+Treg cells as a prognostic marker in pancreatic cancer.

In our meta-analysis, 8 studies involving 972 patients were analyzed. The HRs for the association between intratumoral FoxP3+Treg cell level and OS and the indicators of recurrence (DFS/PFS/RFS) are 2.13 and 1.70, respectively. Similarly, the HRs for the association between the peritumoral FoxP3+Treg cell level and OS is 2.17. These pooled results showed that a high density of tumor-infiltrating FoxP3+Treg cells are associated with poor survival and high recurrence, regardless of whether they are found in the intratumoral or peritumoral tissue of pancreatic cancer. The results are consistent with the initial hypothesis that FoxP3+Treg cells inhibit anti-tumor immunity. Tumor-infiltrating lymphocytes in our meta-analysis refer to the lymphocytes infiltrating the tumor bed but not peripheral circulating blood. This is more likely to reflect the tumor’s immune microenvironment and the interaction of immune cells. These results are potentially important for the prognosis and treatment of pancreatic cancer.

The prognosis of patients with pancreatic cancer is very poo r[41, 42]. The NEJM [43] and other magazines have reported that the monoclonal antibody drug blocking immunoassay (such as PD-1 and PD-L1) is effective for treating non-small cell lung cancer, malignant melanoma, renal cell carcinoma, ovarian cancer, stomach cancer, and breast cancer. However, the effect of the treatment effect in pancreatic cancer was not reported. This may be because there was little or no infiltration of cytotoxic T cells in pancreatic cancer tissue.

One of the possible reasons for this finding is that FoxP3+Treg cells suppressed the function of cytotoxic T cells to destroy the function of anti-tumor, which resulted in the escape of immunological surveillance [44]. It is reported FoxP3+Treg cells secrete TGF-β, which means that the suppression of anti-tumor immunity of FoxP3+Treg cells may be cytokine-dependent [45]. FoxP3+Treg cells also secreted IL-10 to suppress Th1/2 cell proliferation and down-regulating MHC class II in monocytes [46]. However, some studies have shown that FoxP3+Treg down-modulate immune function by generates adenosine. These mechanisms may account for the poor survival outcomes in pancreatic cancer patients with high expression of FoxP3+Treg cells.

The initial view that FoxP3+Treg cells always suppress tumor immunity was challenged in the case of gastrointestinal tumors. The discrepancy in some studies may result from different research methods or the biological characteristics of specific tumor types. We need to better understand the function of FoxP3+Treg cells and their different biological characteristics in the tumor. Our study elucidates the effect of FoxP3+Treg on patient prognosis in the pancreatic cancer tumor microenvironment. Meta-analysis is useful to integrate the results from all single studies for an uncertain outcom e[47]. In addition, we also innovatively studied the influence of FoxP3+Treg cell infiltration in different spatial sites on prognosis. Overall survival (OS) and disease-free survival (DFS)/progression-free survival (PFS)/relapse-free survival (RFS) were investigatory, and the study has therefore been very comprehensive and thorough.

However, due to the limitations of our study, we should be careful when dealing with these results. There are several limitations that need to be considered. First, this study was constrained to studies published in the English language, and we may be missing studies published in other languages. Second, the patients from the 8 studies included in our analysis did not necessarily have consistent treatments and the category of TNM and histologic types of pancreatic cancer varied. Fortunately, the sensitivity analysis showed that individual studies had little effect on the overall outcome. Third, although the data indicate that there is no significant publication bias in the studies that used OS as a survival outcome, the potential publication bias was unavoidable and some data could still be missing. Fourth, experimental reagents, immunohistochemical scoring strategies, and truncated values were not exactly the same in all 8 studies. Finally, the HR and 95% CIs extracted from survival curves but directly obtained from the articles may be less reliable.

## Conclusion

Our meta-analysis based on currently published articles indicated that a higher level of FoxP3+Treg cells was associated with poor overall survival and recurrence. Accordingly, FoxP3+Treg cells can be considered as a promising therapeutic target in pancreatic cancer.

## Data Availability

The datasets analyzed for the present study are available from the corresponding author upon reasonable request.
